# [^18^F]PSMA-1007 PET for biochemical recurrence of prostate cancer, a comparison with [^18^F]Fluciclovine

**DOI:** 10.1186/s41824-024-00228-2

**Published:** 2024-11-27

**Authors:** Cato C. Loeff, Willemijn van Gemert, Bastiaan M. Privé, Inge M. van Oort, Rick Hermsen, Diederik M. Somford, James Nagarajah, Linda Heijmen, Marcel J. R. Janssen

**Affiliations:** 1 Department of Radiology and Nuclear Medicine, Radboud University Medical Center, Radboud Institute for Health Sciences, PO Box 9101, 6500 HB Nijmegen, The Netherlands; 2 Department of Radiation Oncology, Erasmus Medical Center, Cancer Institute, Rotterdam, The Netherlands; 3 Department of Urology, Radboud University Medical Center, Radboud Institute for Health Sciences, Nijmegen, The Netherlands; 4 Department of Nuclear Medicine, Canisius Wilhelmina Hospital, Nijmegen, The Netherlands; 5 Department of Urology, Canisius Wilhelmina Hospital, Nijmegen, The Netherlands; 6 Department of Radiology and Nuclear Medicine, Leiden University Medical Center, Leiden, The Netherlands

**Keywords:** PET/CT, PSMA, Fluciclovine, Biochemical recurrence, Prostate cancer (PCa)

## Abstract

**Aim:**

The objective of this study was to compare the detection rates of [^18^F]PSMA-1007 and [^18^F]Fluciclovine in early biochemical recurrence (BCR) of prostate cancer, i.e. with low prostate-specific antigen (PSA) levels (0.2–5.0 µg/L).

**Methods:**

This was a prospective, single-center (Radboudumc; Nijmegen, The Netherlands), comparative phase II diagnostic imaging study (NCT04239742). The main inclusion criteria were histologically proven adenocarcinoma of the prostate, BCR after radical treatment with two consecutive (rising) PSA values (0.2–5.0 µg/L). Patients underwent both [^18^F]PSMA-1007 PET/CT and [^18^F]Fluciclovine PET/CT within two weeks. Both scans were blindly scored by three independent nuclear medicine physicians. Hereafter, a result per scan and region was generated by consensus. The primary outcome was to compare the detection rate on a patient and region level. Secondary objectives were to determine detection rate stratified for PSA value, inter-reader agreement, and SUV measurements. For lesion confirmation a composite reference score was established using follow-up data.

**Results:**

Data of fifty patients were included, median age of 71 (IQR: 67–74) years and median PSA value of 0.38 (IQR: 0.30–1.55) µg/L. Detection rates were 68% (34/50) for [^18^F]PSMA-1007 and 42% (21/50) for [^18^F]Fluciclovine on a patient level (*p* < 0.001). Detection rates stratified for PSA value of [^18^F]PSMA-1007 in comparison with [^18^F]Fluciclovine were for PSA 0.2–0.5 µg/L; 60.7% versus 25.0% (*p *= 0.002); and for PSA ≥ 0.5 µg/L; 77.3% versus 63.6% (*p *= 0.250). There was a trend for higher inter-reader agreement with [^18^F]PSMA-1007. SUV_max_ (*p* < 0.001) was significantly higher for [^18^F]PSMA-1007 in comparison to [^18^F]Fluciclovine.

**Conclusion:**

In patients with early BCR of prostate cancer after radical surgery or radiotherapy, [^18^F]PSMA-1007 demonstrated a significantly higher detection rate than [^18^F]Fluciclovine. This is particularly relevant since earlier and more accurate detection of a BCR can guide salvage therapy into a tailored strategy which may improve outcomes.

*Trial registration*: ClinicalTrials.gov, NCT 04239742. Registered 02 January 2020, https://clinicaltrials.gov/study/NCT04239742.

**Supplementary Information:**

The online version contains supplementary material available at 10.1186/s41824-024-00228-2.

## Introduction

Prostate cancer is the most common non-skin cancer in men worldwide (Ferlay et al. 2018). After radical surgery or radiotherapy (EBRT) approximately 27–53% of patients develops biochemical recurrence (BCR) (Cornford et al. 2021; Chung et al. 2021). At low prostate-specific antigen (PSA) values, there is a high chance of having a local recurrence or oligometastatic disease and therefore eligibility for salvage treatment (Ost et al. 2018; Kim et al. 2017). To localize the recurrent disease, positron-emission tomography/computed tomography (PET/CT) is favored over Magnetic Resonance Imaging (MRI) and bone scintigraphy, due to its higher diagnostic accuracy, especially at lower PSA concentration (≤ 2.0 µg/L) (Cornford et al. 2021; McCormick et al. 2019; Kane et al. 2003).

To date, several PET tracers for prostate cancer have been developed. In 2016, the Food and Drug Administration (FDA) approved the synthetic amino acid labeled with Fluor-18 [^18^F]Fluciclovine (anti-1-amino-3-^18^F-fluorocyclobutane-1-carboxylic acid) (Axumin) for prostate cancer recurrence as it showed superior sensitivity and specificity compared to conventional imaging modalities (Kane et al. 2003; Chen et al. 2019; Administration 2016). Fluciclovine is recognized and internalized into the cell by amino acid transporters, that are upregulated on the prostate cancer cell surface due to the increased energy demand (Oka et al. 2012). Other PET tracers for prostate cancer target the prostate-specific membrane antigen (PSMA). PSMA is an amino acid type II transmembrane glycoprotein. This is highly over-expressed by the majority of prostate cancer cells (Silver et al. 1997; Wright et al. 1995). Based on their mode of action, PSMA tracers are suggested to be favored in detecting prostate cancer recurrence (Fendler et al. 2019; Evans et al. 2018). One widely used PSMA tracer is Gallium-68-labeled [^68^Ga] Glu-urea-Lys (Ahx)-HBED-CC (PSMA-11) (Hope et al. 2019), reported with a detection rate around 50.5–57.9% in the low PSA value range (0.2–0.5 µg/L) (Abghari-Gerst et al. 2022; Eiber et al. 2015).

Recently, a novel ^18^F labelled PSMA tracer was developed called: [^18^F]PSMA-1007 (Giesel et al. 2017; Cardinale et al. 2017). The ligand PSMA-1007 is primary cleared by the hepatobiliary tract instead of via the urinary tract. The low renal excretion improves the detection of tumor lesions in proximity of urethra, bladder and ureters, where most recurrences occur (Giesel et al. 2019). Also, compared to ^68^Ga, the radionuclide ^18^F has several advantages. The daily available activity of generator produced ^68^Ga is limited compared to ^18^F which is generated in a cyclotron. ^18^F also has a longer half-life compared to ^68^Ga (109 vs 68 min), and is thus more practical for centralized production and distribution (Kesch et al. 2017). Moreover, the longer half-life of ^18^F enables imaging at a later time point, that improves internalization of PSMA into tumor lesions and therefore the lesion-to-background ratio (Sahlmann et al. 2016). Furthermore, the lower positron energy of ^18^F results in shorter positron range and therefore provides higher resolution images. Previous studies suggest that [^18^F]PSMA-1007 may lead to improved detection rates in BCR, especially at low serum PSA levels (Mingels et al. 2022; Olivier et al. 2023).

A study, released recently, comparing [^18^F]Fluciclovine and [^68^Ga]Ga-PSMA-11, showed improved biochemical recurrent prostate cancer detection with [^68^Ga]Ga-PSMA-11 (Calais et al. 2019). However, no such study is presently done for [^18^F]PSMA-1007. We therefore performed a prospective directly comparative study to investigate the detection rate of [^18^F]PSMA-1007 and [^18^F]Fluciclovine in patients with early BCR of prostate cancer, with low PSA levels, i.e. 0.2–5.0 µg/L.

## Materials and methods

This was a prospective, single center (Radboudumc; Nijmegen, The Netherlands), comparative phase II diagnostic imaging study. The study was conducted according to the principles of the Declaration of Helsinki (version 2013) and approved by the medical ethical committee Arnhem-Nijmegen. All patients provided written informed consent. This study was registered on ClinicalTrials.gov, NCT 04239742.

### Patients

The study included adult men with histologically proven adenocarcinoma of the prostate and BCR (PSA 0.2–5.0 µg/L) after one or more treatments with curative intent. Two consecutive (rising) PSA values were required and the most recent PSA value must be obtained within 8 weeks before study participation. The study aimed to include at least half of the patients (>25) with a PSA value 0.2–0.5 µg/L. Patients were excluded if they had a second cancer within two years prior to BCR or if they had a contra-indication for PET/CT imaging.

### Procedures

All patients had [^18^F]PSMA-1007 PET/CT and [^18^F]Fluciclovine PET/CT at least 24 h apart within 2 weeks in random order. Preparation of both tracers was done according to standardized procedures as prescribed by the provider. All patients received routine PET/CT from head to mid-femoral with the first bed position being the pelvic region (Imaging procedures and maximum standardized uptake value (SUV_max_) assessment, Online Resource 1). Adverse events (AEs) were assessed by telephonic interviews within three to five days after the scan. For analysis, all scans were anonymized. Both scans were randomly and blindly scored by three independent nuclear medicine physicians (Assessment Form, Online Resource 2). The readers were not aware of patient characteristics, such as medical history and recent PSA values. Readers assessed the presence of prostate cancer for seven regions according to TNM classification: prostate (bed) (T), pelvic lymph nodes left and right (N), distant lymph nodes (M1a), skeletal lesions (M1b), visceral lesions (M1c), or other distant lesions (M1x). Level of suspicion (LOS) was scored on a 5-point scale following PSMA-RADS (Rowe et al. 2018): (1) probably benign, (2) uncertain benign, (3) uncertain, (4) uncertain malignant, (5) probably malignant. Presence of prostate cancer was defined negative (LOS 1-2), unsure (LOS 3) or positive (LOS4-5) by each reader. Hereafter, a consensus reading was performed classifying the scan as a whole and regions as either positive or negative based on LOS, location, absence or presence of corresponding substrate on CT, and SUV_max_ (Consensus scoring form, Online Resource 3).

### Lesion validation

All available disease related follow-up data, including PSA changes after local/targeted treatment, imaging and histology, was attained to form a composite reference score for lesion validation. Lesion validation was done per region (prostate (bed), pelvic lymph nodes, distant lymph nodes, skeletal lesions, visceral lesions, or other distant lesions). PET-positive lesions that could be validated per region by histopathology, progression on additional or follow-up imaging, or PSA decrease (> 50%) after local/targeted treatment were found true positive (TP). Progression of lesions on additional or follow-up imaging was defined as increase in size, increase in activity accumulation, increased substrate, suspicious location, or a combination thereof. In case of a PET-positive lesion with a negative follow-up scan without intervention, negative histopathology, PSA decrease of < 50% after local/targeted treatment, or PSA decrease without intervention, lesions were accounted per region as false positives (FP). PET-negative lesions which remained negative on follow-up imaging or showed no response to PSA after local/targeted treatment were scored as true negative (TN) per region. Because PSA rise is indicative of recurrent disease, all negative PET-scans were scored as false negative (FN) as a whole.

### Outcomes

The primary outcome was to compare the detection rates of [^18^F]PSMA-1007 and [^18^F]Fluciclovine. Detection rate was defined as the number of PET-positive scans detected per patient (scan as a whole) and per region, independent of follow-up.

Secondary objectives were to compare the detection rates stratified by PSA value (0.2–0.5 vs ≥ 0.5 µg/L), to evaluate the inter-reader agreement for both tracers, and to compare SUV measurements for both tracers in the subset of patients with at least one PET-positive lesion.

### Statistical analysis

A statistical power analysis found that a sample size of fifty patients provided a power of at least 80% to detect the expected 20% detection rate difference at the patient level in favor of [^18^F]PSMA-1007 PET/CT. Baseline characteristics were described with descriptive statistics. For each tracer was scored whether disease activity (PET-positive lesion(s)) was detected in each patient as a whole, and also for each anatomical region (prostate (bed), pelvic lymph nodes, distant lymph nodes, bones or other organs (categorical data: either PET-positive lesion(s) were detected or not detected). The detection rate on a patient and region level were compared using a two-sided McNemar’s test for paired proportions. The same analyses were performed stratified for PSA concentrations (0.2–0.5 vs ≥ 0.5 µg/L). Fleiss multirater κ statistics were performed to assess inter-reader agreement between readers for both tracers. Κ values less than 0.00 are defined as poor disagreement, 0.00–0.40 as slight to fair agreement, 0.41–0.60 indicate moderate agreement, and 0.61–1.00 indicates substantial to high agreement above the chance of agreement (Landis and Koch 1977). The Wilcoxon signed-rank test was used to compare SUV_max_ of tumor lesions. The statistical analyses were performed with SPSS version 25 and Graphpad version 9.0.0.

## Results

### Demographics

Fifty-two patients were enrolled between January 30, 2020 and June 8, 2021. Two patients were excluded: one patient received only one scan because the second was not allowed due to COVID-19 regulations and the other patient had a second malignancy within two years of BCR. Resulting in a total of fifty cases that were available for analyses. Demographics and clinical characteristics of these fifty patients are presented in Table 1. The median age was 71 (IQR: 67–74) years. Median PSA value at enrollment was 0.38 (IQR: 0.30–1.55) µg/L and median PSA doubling time was 5.3 (IQR: 2.5–11.4) months. Twenty-eight (56%) patients were in the PSA category 0.2–0.5 µg/L. The median Gleason score was 7 (IQR: 7–8) and the median initial PSA (iPSA) at diagnosis was 8.1 (IQR: 6.0–16.0) µg/L. PET/CT scan characteristics are provided in Online Resource 4. No AEs were reported that seemed related to both tracers.

**Table 1:** Demographic and clinicopathological characteristics, n = 50. Presented as median (interquartile range) or frequencies (percentage)

Age, (years)	71 (IQR: 67–74)
Gleason score	7 (IQR: 7–8)
ISUP 1	1 (2%)
ISUP 2	25 (50%)
ISUP 3	8 (16%)
ISUP 4	9 (18%)
ISUP 5	7 (14%)
PSA at initial diagnosis, µg/L	8.10 (IQR: 6.0–16.0)
PSA doubling time, months	5.3 (IQR: 2.5–11.4)
Last PSA value, µg/L	0.38 (IQR: 0.30–1.55)
0.20–0.30	12 (24%)
0.30–0.40	14 (28%)
0.40–0.50	2 (4%)
≥ 0.5	22 (44%)
Histopathological TNM stage	
Primary tumor staging	
T1	1 (2%)
T2	19 (38%)
T3	30 (60%)
Lymph node staging	
N0	26 (52%)
N1, histologically proven	9 (18%)
Nx	15 (30%)
Distant metastases	
M0	50 (100%)
Previous therapy	
Radical prostatectomy	41 (82%)
Surgical margin positive	15 (30%)
Local radiotherapy	19 (38%)
Lymph node dissection	27 (54%)
Adjuvant Androgen deprivation therapy*	7 (14%)

Data are n (%) or median (IQR). TNM classification was divided in clinical (n = 5) and pathological (n = 45)

PSA, prostate-specific antigen

*Only temporary additional hormonal treatment in adjuvant setting to reduce risk of disease recurrence.

### Scan performances

**Fig. 1:**
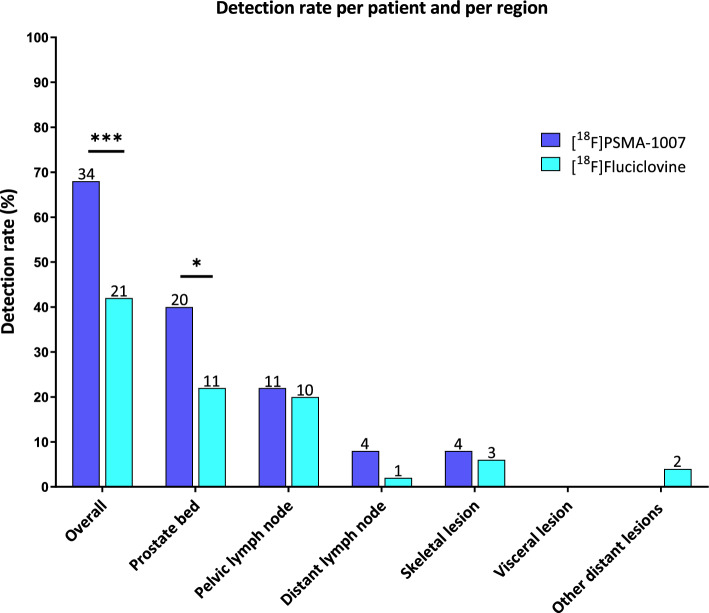
Detection rate per region and per patient for [^18^F]PSMA-1007 PET/CT and [^18^F]Fluciclovine PET/CT. Two-sided McNemars test. Significant if *p* ≤ 0.05. * indicates *p* ≤ 0.05 and *** indicates *p* ≤ 0.001. The number on top of the bar indicates the number of patients

Thirty-four (68%) of [^18^F]PSMA-1007 PET/CT scans were scored positive whereas 21 (42%) of [^18^F]Fluciclovine PET/CT scans were scored positive (Fig. 1 and Online Resource 5). Sixteen (32%) patients were scored negative in both tracers. Twenty-one (42%) patients were scored positive in both tracers. Thirteen (26%) patients were scored positive with [^18^F]PSMA-1007 but negative with [^18^F]Fluciclovine. No scans were positive with [^18^F]Fluciclovine and negative with [^18^F]PSMA-1007 (Online Resource 6).

The detection rate per patient was significantly higher for [^18^F]PSMA-1007 (*p* = < 0.001) (Online Resource 5 and 6). The additional lesions observed with [^18^F]PSMA-1007 were located in prostate (bed) region (T), pelvic lymph nodes (N), distant lymph nodes (M1a), and skeletal lesions (M1b). In particular, [^18^F]PSMA-1007 PET/CT showed significantly more lesions in the prostate (bed) compared to [^18^F]Fluciclovine PET/CT (*p* = 0.022) (Fig. 1, Online Resource 5 and 7). No statistically significant differences were observed for the pelvic lymph nodes (N), distant lymph nodes (M1a), and other distant lesions (M1x). In four (8%) and three (6%) patients skeletal lesions were observed with [^18^F]PSMA-1007 and [^18^F]Fluciclovine, respectively. No visceral lesions (M1c) were detected with either tracer.

**Fig. 2:**
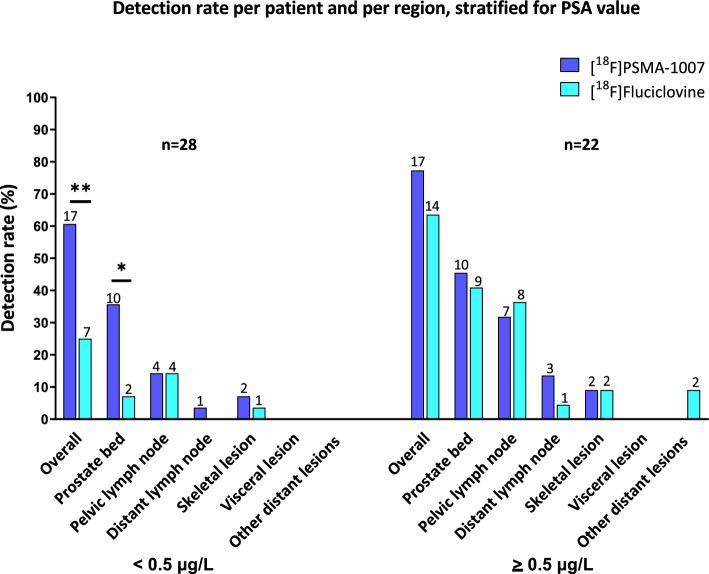
Detection rate per patient and per region for [^18^F]PSMA-1007 PET/CT and [^18^F]Fluciclovine PET/CT in relation to PSA levels (0.2–0.5 µg/L vs ≥ 0.5 µg/L). Two-sided McNemars test. Significant if *p* ≤ 0.05. * indicates *p* ≤ 0.05, ** indicates *p* ≤ 0.010. The number on top of the bar indicates the number of patients

Detection rates per patient and per region stratified for PSA value are shown in Fig. 2 and provided in Online Resource 8. For both tracers, the detection rate increases with higher PSA levels. Overall detection rate on a per patient level in the PSA range 0.2–0.5 µg/L (n = 28) was 17 (60.7%) versus seven (25.0%) for [^18^F]PSMA-1007 and [^18^F]Fluciclovine, respectively (*p *= 0.002). [^18^F]PSMA-1007 showed a higher detection rate in the prostate (bed) region in this low PSA range (*p* = 0.021). In the PSA range ≥ 0.5 µg/L (n = 22), the detection rates for [^18^F]PSMA-1007 and [^18^F]Fluciclovine, were not significantly different (*p* = 0.250).

Inter-reader agreement was based on the three reader sets (Online Resource 9). Inter-reader agreement for both tracers in all regions was indicated as moderate agreement (k values of 0.41–0.60). Although it includes values that overlap, there is a higher trend for [^18^F]PSMA-1007 in comparison with [^18^F]Fluciclovine for prostate (bed) region and lymph node metastases (Table 2).

**Table 2:** Inter-reader measures of agreement for lesions in different regions

Region	[^18^F]PSMA-1007 PET/CT	[^18^F]Fluciclovine PET/CT
Prostate (bed)	0.59 (0.46–0.72)	0.49 (0.37–0.61)
Lymph node metastases	0.73 (0.63–0.82)	0.59 (0.50–0.68)
Hematologic metastases	0.52 (0.44–0.61)	0.57 (0.48–0.66)

The Fleiss multirater κ statistics (confidence interval) was used to assess inter-reader agreement between the readers for each tracer ([^18^F]PSMA-1007 and [^18^F]Fluciclovine) for lesions in different regions. Lymph node metastases include pelvic and distant lymph nodes. Hematologic metastases include skeletal and visceral lesions. A positive number of κ statistics is the degree of agreement above the chance of agreement. Negative κ-statistics indicate that there is less agreement than expected by chance

### Scan outcomes

**Fig. 3:**
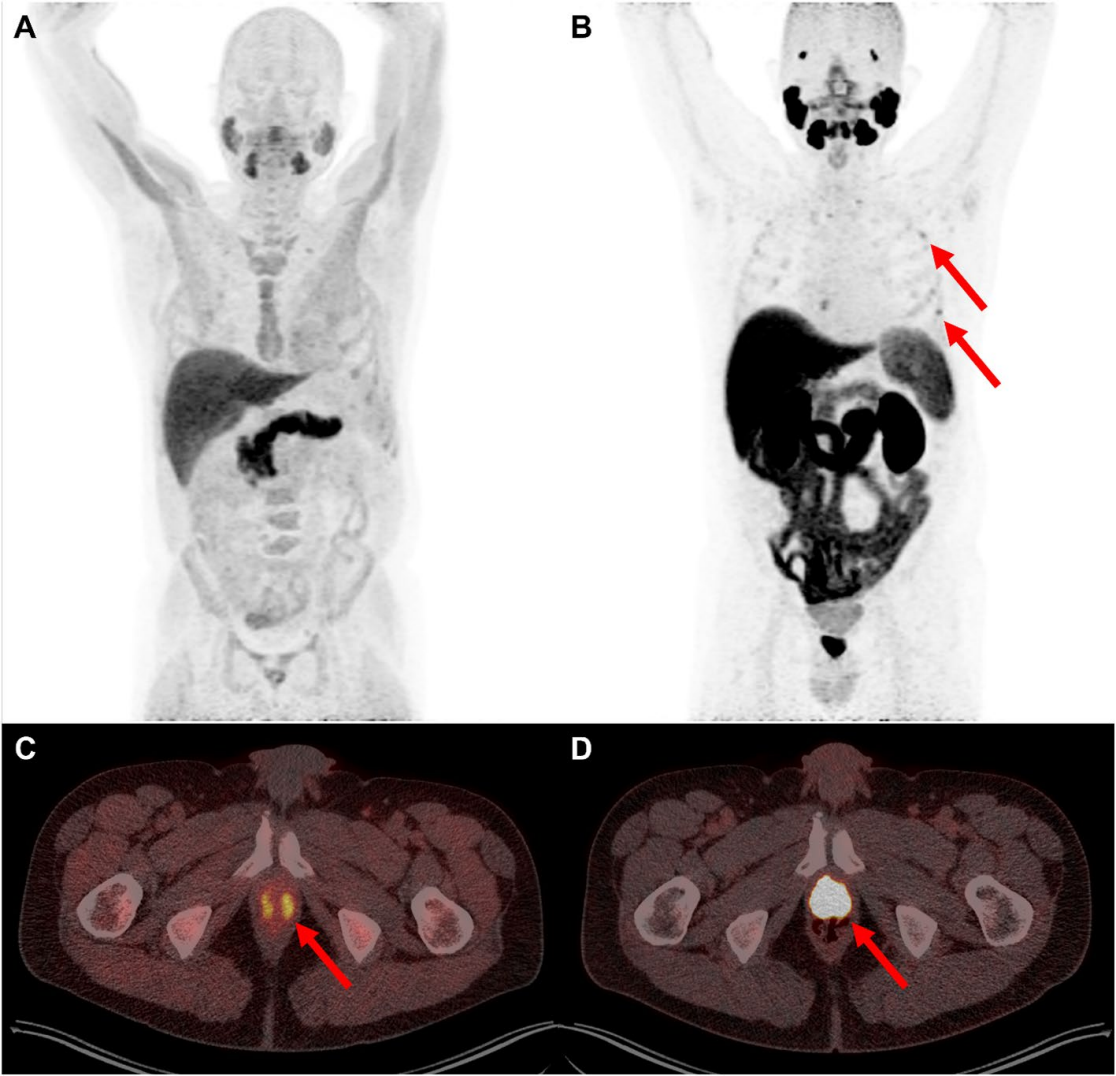
Scans with both tracers of same patient with previous therapy radical prostatectomy. [^18^F]PSMA-1007 PET/CT detecting lesion in prostate (bed) region with higher SUV_max_ than [^18^F]Fluciclovine PET/CT. [^18^F]Fluciclovine PET maximum intensity projection (MIP) image (**a**), no uptake in the ribs is detected. High background uptake in the bone marrow, muscles and pancreas. [^18^F]PSMA-1007 PET MIP image (**b**), with examples of unspecific low level bone uptake in the ribs (red arrows). Transversal slides showing lesion in the prostate (bed) region detected with [^18^F]Fluciclovine PET/CT (SUV_max_ 8.6) (**c**) and [^18^F]PSMA-1007 PET/CT (SUV_max_ 59.6) (**d**)

Twenty-five lesions could be matched for both tracers. The median SUV_max_ of these lesions was significantly higher for [^18^F]PSMA-1007 compared to [^18^F]Fluciclovine as is shown in Table 3 and Fig. 3.

**Table 3:** Median SUV_max_ for [^18^F]PSMA-1007 and [^18^F]Fluciclovine for different lesions

	[^18^F]PSMA-1007 PET/CT	[^18^F]Fluciclovine PET/CT	*p* value
SUV_max_	8.5 (6.3–17.3)	5.7 (4.2–8.3)	<0.001

Median (interquartile range). Wilcoxon signed rank test. Significant if *p *≤ 0.05.

#### Follow-up treatment and lesion validation per region

**Fig. 4:**
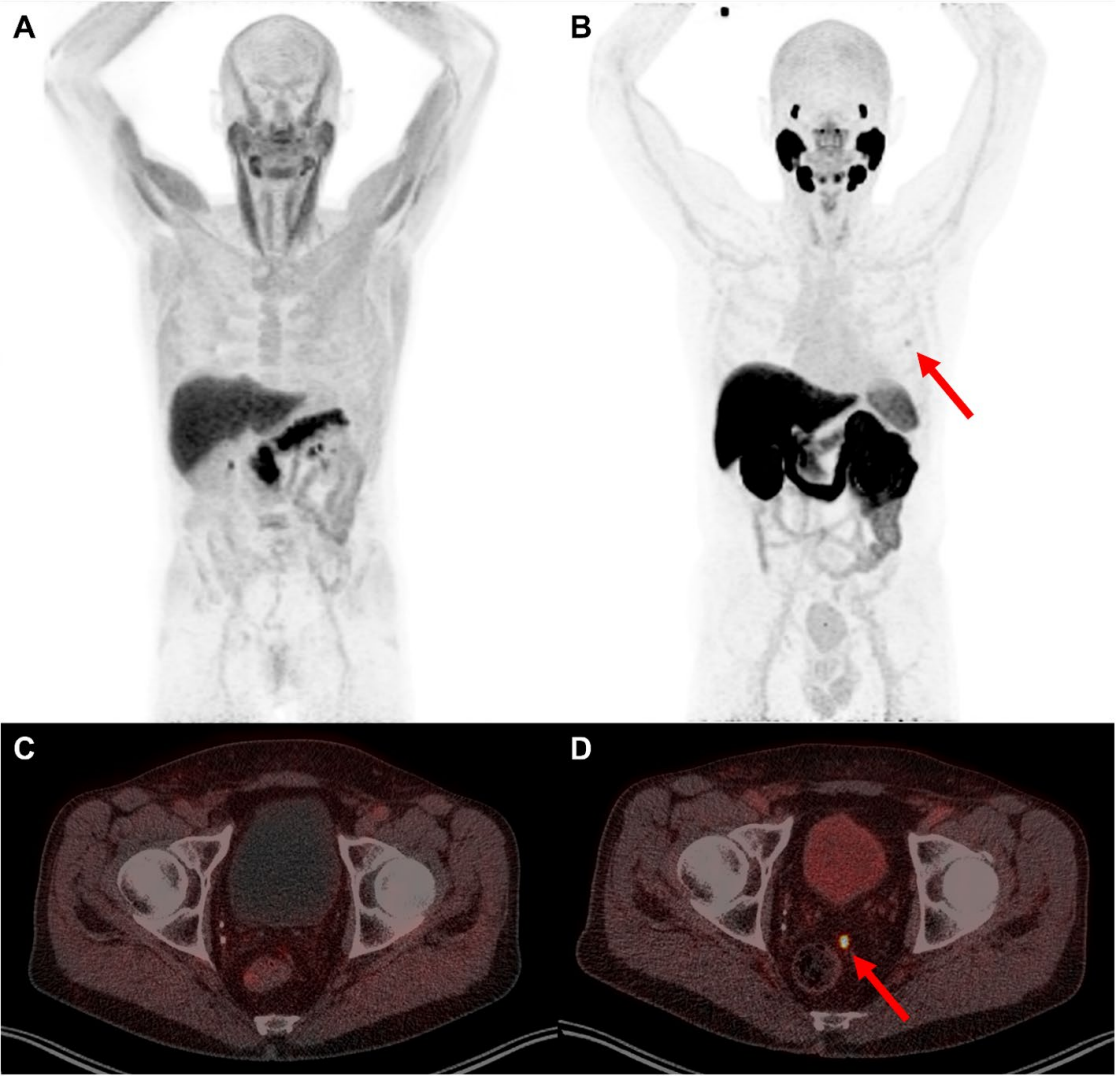
Scans with both tracers of same patient with previous therapy radical prostatectomy, pelvic lymph node dissection and radiation therapy on the prostate (bed). [^18^F]PSMA-1007 PET/CT detecting lesion in pelvic lymph node region and which is not identified with [^18^F]Fluciclovine PET/CT. [^18^F]Fluciclovine PET/CT MIP image (**a**), shows no uptake in the ribs. High background uptake in the bone marrow, muscles and pancreas. [^18^F]PSMA-1007 PET/CT MIP image (**b**) shows slight unspecific bone uptake in a rib (red arrow). Transversal fused PET/CT slides showing no lesions detected with [^18^F]Fluciclovine PET/CT (**c**) and small pelvic lymph node lesion left (red arrow) detected with [^18^F]PSMA-1007 PET/CT (**d**)

Forty-eight (96%) patients had follow-up data available with a median follow-up period of 28.5 (IQR 25.5–29.9) months. Follow-up treatment was available for 46 (92%) patients and is summarized in Online Resource 10. PET-positive findings could not be confirmed per region for 29% (10/34) of [^18^F]PSMA-positive scans and 33% (7/21) of [^18^F]Fluciclovine-positive scans as for example these patients received systemic therapy without previous confirmation of lesions. PET-positive findings were confirmed as TP per region in 62% (21/34) of [^18^F]PSMA-positive scans and 43% (9/21) [^18^F]Fluciclovine-positive scans (Fig. 4). One [^18^F]PSMA-positive suspicious lesion in the prostate (bed) region and one [^18^F]Fluciclovine-positive suspicious lesion in the prostate (bed) region and one in the pelvic lymph nodes region were validated as FP (Online Resource 11). Since not all PET findings could be validated by a gold reference standard, neither sensitivity or specificity could be reliably established.

## Discussion

In this prospective study, that focused on patients with early BCR of prostate cancer, we directly compared the tracers [^18^F]PSMA-1007 and [^18^F]Fluciclovine. In this setting we observed that [^18^F]PSMA-1007 (68%) has a higher detection rate compared to [^18^F]Fluciclovine (42%). Especially in the lower PSA range (0.2–0.5 µg/L), [^18^F]PSMA-1007 outperformed [^18^F]Fluciclovine, which is an important finding as the chance for cure of a salvage intervention is higher at low PSA levels (Trock et al. 2008; Pfister et al. 2014).

The presently observed detection rates of both tracers are within previously reported ranges of 52–60% and 26–56% for [^18^F]PSMA-1007 and [^18^F]Fluciclovine, respectively (Calais et al. 2019; Nanni et al. 2015, 2016; Scarsbrook et al. 2020; Witkowska-Patena et al. 2020; Lengana et al. 2022). However, in most other PSMA imaging studies using [^68^Ga]Ga-PSMA-11 or [^18^F]DCFPyL, higher detection rates of 80–95% are reported (Eiber et al. 2015; Giesel et al. 2019; Ahmadi Bidakhvidi et al. 2021; Grubmüller et al. 2018; Afshar-Oromieh et al. 2015, 2017). The present lower detection rate for [^18^F]PSMA-1007 PET is likely due to the high number of patients with very low PSA levels of 0.2–0.5 µg/L, which is an underreported cohort for PSMA PET scans to date. This is further corroborated by our observation (Fig. 2) that a higher PSA value strongly correlates with a higher detection rate, which was also reported by others (Witkowska-Patena et al. 2020).

In this study, the SUV_max_ of concordant PET-positive lesions was higher for [^18^F]PSMA-1007 in comparison to [^18^F]Fluciclovine (Fig. 3). We postulate that the higher specific radiofarmacon internalization results in a higher SUV_max_ and contributes to the superior detection rate of [^18^F]PSMA-1007 over [^18^F]Fluciclovine. In a region based analysis the difference in detection rate was largest for the prostate (bed), where [^18^F]PSMA-1007 showed more lesions, which were al TP, compared to [^18^F]Fluciclovine. These differences are probably due to the higher SUV_max_ of [^18^F]PSMA-1007 over [^18^F]Fluciclovine and not the low urinary excretion rates of both tracers. There was also a trend for better inter-reader agreement for [^18^F]PSMA-1007 in the prostate (bed) region and lymph node metastases. This is in line with previously published data (Calais et al. 2019; Kuo et al. 2023).

Although a known pitfall of the [^18^F]PSMA-1007 tracer it the unspecific bone uptake, particularly in the ribs (as illustrated in Figs. 3, 4) (Mingels et al. 2022; Ahmadi Bidakhvidi et al. 2021; Rauscher et al. 2020; Grünig et al. 2021; Rizzo et al. 2024), this did not actually translate into a higher detection rate of bone lesions by [18F]PSMA-1007 in our study. Since each PSMA tracer shows different characteristics, training is required when reading these PSMA PET scans (Eiber et al. 2018). SUV_max_ and presence or absence of any correlative lesion on CT are used in distinguishing unspecific bone lesions from justified bone metastases. Our readers assessed most of these bone lesions as negative due to their experience with this tracer, and supported by the follow-up data gathered during this trial. This suggests that if readers are well trained for reading [^18^F]PSMA-1007 scans, this does not result in unwanted over-detection of FP bone lesions. As these FP bone lesions could have impact on therapeutic decisions (Grünig et al. 2021), sophisticated reader training incorporating knowledge about the unspecific lesion distribution and reporting within the clinical context are essential in further clinical implementation of this tracer in hospitals.

The study is limited by its relatively small sample size. Even though our sample size of fifty patients was powered to show a superiority for [^18^F]PSMA-1007, it prevented us from performing post-hoc analyses to stratify for more than two PSA categories, or review the lesions per region. Only in the prostate (bed) region enough lesions were found to detect a statistically significant difference. Moreover, due to the generally low PSA level and thus the small size of the metastases, histopathologic validation lacked in the majority of the results. Therefore, like most other diagnostic studies, we also relied on secondary criteria to complement histopathology, especially the PSA or imaging response on a local/targeted treatment (Olivier et al. 2023; Hofman et al. 2020). Several studies with PSMA-targeting PET tracers have already proven its accuracy for detection of histologically proven metastatic lymph nodes (Rauscher et al. 2016; Herlemann et al. 2016). The design of this study did not enable consideration of the impact of these precise imaging modalities on treatment decisions and patient outcomes. In the EMPIRE-1 trial, where [^18^F]Fluciclovine was incorporated for radiotherapy planning, improved survival free from BCR or persistence was showed (Jani et al. 2021). Also data from PSMA tracers showed promising results on therapeutic decision making, therapy changes, and outcome (Olivier et al. 2023; Bianchi et al. 2023; Pozdnyakov et al. 2023).

All in all, in the light of the results of our comparative data, theoretically the use of [^18^F]PSMA-1007 may yield better outcomes over [^18^F]Fluciclovine. The present study showed that [^18^F]PSMA-1007 outperforms [^18^F]Fluciclovine in overall detection rate and detection rate in the prostate (bed). Also, we postulate that when tumor lesions in close proximity to the urinary tract (e.g. bladder, ureters) are anticipated, low urinary excreting tracers such as [^18^F]PSMA-1007 are recommended. We now await the final results of the pivotal trial of [^18^F]PSMA-1007 (NCT04742361).

## Conclusion

In this prospective directly comparative study in prostate cancer patients with early BCR, [^18^F]PSMA-1007 showed a superior detection rate over [^18^F]Fluciclovine. Earlier and more accurate detection of a local recurrence and/or (oligo)metastatic lesions can guide salvage therapy into a tailored strategy which may improve outcomes.

## Supplementary Information

Additional file 1

Additional file 2

Additional file 3

Additional file 4

Additional file 5

Additional file 6

Additional file 7

Additional file 8

Additional file 9

Additional file 10

Additional file 11

## Data Availability

The datasets generated and analysed during the current study are available from the corresponding author on reasonable request.

## Abbreviations

Biochemical recurrence: BCR
Prostate-specific antigen: PSA
Positron-emission tomography/computed tomography: PET/CT
Magnetic resonance imaging: MRI
The food and drug administration: FDA
Fluor-18: [^18^F]
Prostate-specific membrane antigen: PSMA
Gallium-68-labeled: [^68^Ga]
Glu-urea-Lys (Ahx)-HBED-CC: PSMA-11
Adverse events: AEs
Level of suspicion: LOS
True positive: TP
True negative: TN
False positive: FP
False negative: FN
Standardized uptake value maximum: SUV_max_
Initial PSA: IPSA

## References

[CR1] Abghari-Gerst M, Armstrong WR, Nguyen K, Calais J, Czernin J, Lin D et al (2022) A comprehensive assessment of (68)Ga-PSMA-11 PET in biochemically recurrent prostate cancer: results from a prospective multicenter study on 2,005 patients. J Nucl Med 63(4):567–572 34326126 10.2967/jnumed.121.262412 PMC8973291

[CR2] Administration UFD. FDA approves new diagnostic imaging agent to detect recurrent prostate cancer 2016. Available from: https://www.fda.gov/news-events/press-announcements/fda-approves-new-diagnostic-imaging-agent-detect-recurrent-prostate-cancer

[CR3] Afshar-Oromieh A, Avtzi E, Giesel FL, Holland-Letz T, Linhart HG, Eder M et al (2015) The diagnostic value of PET/CT imaging with the (68)Ga-labelled PSMA ligand HBED-CC in the diagnosis of recurrent prostate cancer. Eur J Nucl Med Mol Imaging 42(2):197–209 25411132 10.1007/s00259-014-2949-6 PMC4315487

[CR4] Afshar-Oromieh A, Holland-Letz T, Giesel FL, Kratochwil C, Mier W, Haufe S et al (2017) Diagnostic performance of (68)Ga-PSMA-11 (HBED-CC) PET/CT in patients with recurrent prostate cancer: evaluation in 1007 patients. Eur J Nucl Med Mol Imaging 44(8):1258–1268 28497198 10.1007/s00259-017-3711-7 PMC5486817

[CR5] Ahmadi Bidakhvidi N, Laenen A, Jentjens S, Deroose CM, Van Laere K, De Wever L et al (2021) Parameters predicting [(18)F]PSMA-1007 scan positivity and type and number of detected lesions in patients with biochemical recurrence of prostate cancer. EJNMMI Res 11(1):41 33929626 10.1186/s13550-021-00783-w PMC8087750

[CR6] Bianchi L, Ceci F, Balestrazzi E, Costa F, Droghetti M, Piazza P et al (2023) PSMA-PET guided treatment in prostate cancer patients with oligorecurrent progression after previous salvage treatment. Cancers (Basel) 15(7):2027 37046687 10.3390/cancers15072027 PMC10093227

[CR7] Calais J, Ceci F, Eiber M, Hope TA, Hofman MS, Rischpler C et al (2019) (18)F-fluciclovine PET-CT and (68)Ga-PSMA-11 PET-CT in patients with early biochemical recurrence after prostatectomy: a prospective, single-centre, single-arm, comparative imaging trial. Lancet Oncol 20(9):1286–1294 31375469 10.1016/S1470-2045(19)30415-2 PMC7469487

[CR8] Cardinale J, Schäfer M, Benešová M, Bauder-Wüst U, Leotta K, Eder M et al (2017) Preclinical evaluation of <sup>18</sup>F-PSMA-1007, a New Prostate-Specific Membrane Antigen Ligand for Prostate Cancer Imaging. J Nucl Med 58(3):425–431 27789722 10.2967/jnumed.116.181768

[CR9] Chen B, Wei P, Macapinlac HA, Lu Y (2019) Comparison of 18F-Fluciclovine PET/CT and 99mTc-MDP bone scan in detection of bone metastasis in prostate cancer. Nucl Med Commun 40(9):940–946 31343613 10.1097/MNM.0000000000001051

[CR10] Chung JH, Jeong JY, Lee JY, Song W, Kang M, Sung HH et al (2021) Biochemical recurrence after radical prostatectomy according to nadir prostate specific antigen value. PLoS ONE 16(5):e0249709 33939714 10.1371/journal.pone.0249709 PMC8092790

[CR11] Cornford P, van den Bergh RCN, Briers E, Van den Broeck T, Cumberbatch MG, De Santis M et al (2021) EAU-EANM-ESTRO-ESUR-SIOG guidelines on prostate cancer. Part II-2020 update: treatment of relapsing and metastatic prostate cancer. Eur Urol 79(2):263–282 33039206 10.1016/j.eururo.2020.09.046

[CR12] Eiber M, Maurer T, Souvatzoglou M, Beer AJ, Ruffani A, Haller B et al (2015) Evaluation of hybrid ⁶⁸Ga-PSMA ligand PET/CT in 248 patients with biochemical recurrence after radical prostatectomy. J Nucl Med 56(5):668–674 25791990 10.2967/jnumed.115.154153

[CR13] Eiber M, Herrmann K, Calais J, Hadaschik B, Giesel FL, Hartenbach M et al (2018) Prostate cancer molecular imaging standardized evaluation (PROMISE): proposed miTNM classification for the interpretation of PSMA-ligand PET/CT. J Nucl Med 59(3):469–478 29123012 10.2967/jnumed.117.198119

[CR14] Evans JD, Jethwa KR, Ost P, Williams S, Kwon ED, Lowe VJ, Davis BJ (2018) Prostate cancer-specific PET radiotracers: a review on the clinical utility in recurrent disease. Pract Radiat Oncol 8(1):28–39 29037965 10.1016/j.prro.2017.07.011

[CR15] Fendler WP, Calais J, Eiber M, Flavell RR, Mishoe A, Feng FY et al (2019) Assessment of 68Ga-PSMA-11 PET accuracy in localizing recurrent prostate cancer: a prospective single-arm clinical trial. JAMA Oncol 5(6):856–863 30920593 10.1001/jamaoncol.2019.0096 PMC6567829

[CR16] Ferlay J, Colombet M, Soerjomataram I, Dyba T, Randi G, Bettio M et al (2018) Cancer incidence and mortality patterns in Europe: estimates for 40 countries and 25 major cancers in 2018. Eur J Cancer 103:356–387 30100160 10.1016/j.ejca.2018.07.005

[CR17] Giesel FL, Hadaschik B, Cardinale J, Radtke J, Vinsensia M, Lehnert W et al (2017) F-18 labelled PSMA-1007: biodistribution, radiation dosimetry and histopathological validation of tumor lesions in prostate cancer patients. Eur J Nucl Med Mol Imaging 44(4):678–688 27889802 10.1007/s00259-016-3573-4 PMC5323462

[CR18] Giesel FL, Knorr K, Spohn F, Will L, Maurer T, Flechsig P et al (2019) Detection efficacy of (18)F-PSMA-1007 PET/CT in 251 patients with biochemical recurrence of prostate cancer after radical prostatectomy. J Nucl Med 60(3):362–368 30042163 10.2967/jnumed.118.212233 PMC6424235

[CR19] Grubmüller B, Baltzer P, D’Andrea D, Korn S, Haug AR, Hacker M et al (2018) (68)Ga-PSMA 11 ligand PET imaging in patients with biochemical recurrence after radical prostatectomy—diagnostic performance and impact on therapeutic decision-making. Eur J Nucl Med Mol Imaging 45(2):235–242 29075832 10.1007/s00259-017-3858-2 PMC5745568

[CR20] Grünig H, Maurer A, Thali Y, Kovacs Z, Strobel K, Burger IA, Müller J (2021) Focal unspecific bone uptake on [(18)F]-PSMA-1007 PET: a multicenter retrospective evaluation of the distribution, frequency, and quantitative parameters of a potential pitfall in prostate cancer imaging. Eur J Nucl Med Mol Imaging 48(13):4483–4494 34120201 10.1007/s00259-021-05424-x PMC8566387

[CR21] Herlemann A, Wenter V, Kretschmer A, Thierfelder KM, Bartenstein P, Faber C et al (2016) (68)Ga-PSMA positron emission tomography/computed tomography provides accurate staging of lymph node regions prior to lymph node dissection in patients with prostate cancer. Eur Urol 70(4):553–557 26810345 10.1016/j.eururo.2015.12.051

[CR22] Hofman MS, Lawrentschuk N, Francis RJ, Tang C, Vela I, Thomas P et al (2020) Prostate-specific membrane antigen PET-CT in patients with high-risk prostate cancer before curative-intent surgery or radiotherapy (proPSMA): a prospective, randomised, multicentre study. Lancet 395(10231):1208–1216 32209449 10.1016/S0140-6736(20)30314-7

[CR23] Hope TA, Goodman JZ, Allen IE, Calais J, Fendler WP, Carroll PR (2019) Metaanalysis of (68)Ga-PSMA-11 PET accuracy for the detection of prostate cancer validated by histopathology. J Nucl Med 60(6):786–793 30530831 10.2967/jnumed.118.219501 PMC6581235

[CR24] Jani AB, Schreibmann E, Goyal S, Halkar R, Hershatter B, Rossi PJ et al (2021) (18)F-fluciclovine-PET/CT imaging versus conventional imaging alone to guide postprostatectomy salvage radiotherapy for prostate cancer (EMPIRE-1): a single centre, open-label, phase 2/3 randomised controlled trial. Lancet 397(10288):1895–1904 33971152 10.1016/S0140-6736(21)00581-X PMC8279109

[CR25] Kane CJ, Amling CL, Johnstone PA, Pak N, Lance RS, Thrasher JB et al (2003) Limited value of bone scintigraphy and computed tomography in assessing biochemical failure after radical prostatectomy. Urology 61(3):607–611 12639656 10.1016/s0090-4295(02)02411-1

[CR26] Kesch C, Kratochwil C, Mier W, Kopka K, Giesel FL (2017) (68)Ga or (18)F for prostate cancer imaging? J Nucl Med 58(5):687–688 28408526 10.2967/jnumed.117.190157

[CR27] Kim J, Park JS, Ham WS (2017) The role of metastasis-directed therapy and local therapy of the primary tumor in the management of oligometastatic prostate cancer. Investig Clin Urol 58(5):307–316 28868501 10.4111/icu.2017.58.5.307 PMC5577326

[CR28] Kuo PH, Yoo DC, Avery R, Seltzer M, Calais J, Nagarajah J et al (2023) A VISION substudy of reader agreement on (68)Ga-PSMA-11 PET/CT scan interpretation to determine patient eligibility for (177)Lu-PSMA-617 radioligand therapy. J Nucl Med 64(8):1259–1265 37230533 10.2967/jnumed.122.265077 PMC10394308

[CR29] Landis JR, Koch GG (1977) The measurement of observer agreement for categorical data. Biometrics 33(1):159–174 843571

[CR30] Lengana T, Lawal I, Janse Van Rensburg C, Mokoala K, Moshokoa E, Mazibuko S et al (2022) The diagnostic performance of 18F-PSMA-1007 PET/CT in prostate cancer patients with early recurrence after definitive therapy with a PSA < 10 ng/ml. Nuklearmedizin 61(2):120–129 35421900 10.1055/a-1759-1603

[CR31] McCormick BZ, Mahmoud AM, Williams SB, Davis JW (2019) Biochemical recurrence after radical prostatectomy: current status of its use as a treatment endpoint and early management strategies. Indian J Urol 35(1):6–17 30692719 10.4103/iju.IJU_355_18 PMC6334583

[CR32] Mingels C, Bohn KP, Rominger A, Afshar-Oromieh A, Alberts I (2022) Diagnostic accuracy of [(18)F]PSMA-1007 PET/CT in biochemical recurrence of prostate cancer. Eur J Nucl Med Mol Imaging 49(7):2436–2444 35067735 10.1007/s00259-022-05693-0 PMC9165245

[CR33] Nanni C, Schiavina R, Brunocilla E, Boschi S, Borghesi M, Zanoni L et al (2015) 18F-fluciclovine PET/CT for the detection of prostate cancer relapse: a comparison to 11C-choline PET/CT. Clin Nucl Med 40(8):e386–e391 26053708 10.1097/RLU.0000000000000849

[CR34] Nanni C, Zanoni L, Pultrone C, Schiavina R, Brunocilla E, Lodi F et al (2016) (18)F-FACBC (anti1-amino-3-(18)F-fluorocyclobutane-1-carboxylic acid) versus (11)C-choline PET/CT in prostate cancer relapse: results of a prospective trial. Eur J Nucl Med Mol Imaging 43(9):1601–1610 26960562 10.1007/s00259-016-3329-1

[CR35] Oka S, Okudaira H, Yoshida Y, Schuster DM, Goodman MM, Shirakami Y (2012) Transport mechanisms of trans-1-amino-3-fluoro[1-(14)C]cyclobutanecarboxylic acid in prostate cancer cells. Nucl Med Biol 39(1):109–119 21958853 10.1016/j.nucmedbio.2011.06.008

[CR36] Olivier P, Giraudet AL, Skanjeti A, Merlin C, Weinmann P, Rudolph I et al (2023) Phase III study of (18)F-PSMA-1007 versus (18)F-Fluorocholine PET/CT for localization of prostate cancer biochemical recurrence: a prospective, randomized. Crossover multicenter study. J Nucl Med 64(4):579–585 36418170 10.2967/jnumed.122.264743 PMC10071780

[CR37] Ost P, Reynders D, Decaestecker K, Fonteyne V, Lumen N, De Bruycker A et al (2018) Surveillance or metastasis-directed therapy for oligometastatic prostate cancer recurrence: a prospective, randomized, multicenter phase II trial. J Clin Oncol 36(5):446–453 29240541 10.1200/JCO.2017.75.4853

[CR38] Pfister D, Bolla M, Briganti A, Carroll P, Cozzarini C, Joniau S et al (2014) Early salvage radiotherapy following radical prostatectomy. Eur Urol 65(6):1034–1043 23972524 10.1016/j.eururo.2013.08.013

[CR39] Pozdnyakov A, Kulanthaivelu R, Bauman G, Ortega C, Veit-Haibach P, Metser U (2023) The impact of PSMA PET on the treatment and outcomes of men with biochemical recurrence of prostate cancer: a systematic review and meta-analysis. Prostate Cancer Prostat Dis 26(2):240–248 10.1038/s41391-022-00544-3 35440642

[CR40] Rauscher I, Maurer T, Beer AJ, Graner FP, Haller B, Weirich G et al (2016) Value of 68Ga-PSMA HBED-CC PET for the assessment of lymph node metastases in prostate cancer patients with biochemical recurrence: comparison with histopathology after salvage lymphadenectomy. J Nucl Med 57(11):1713–1719 27261524 10.2967/jnumed.116.173492

[CR41] Rauscher I, Krönke M, König M, Gafita A, Maurer T, Horn T et al (2020) Matched-pair comparison of (68)Ga-PSMA-11 PET/CT and (18)F-PSMA-1007 PET/CT: frequency of pitfalls and detection efficacy in biochemical recurrence after radical prostatectomy. J Nucl Med 61(1):51–57 31253741 10.2967/jnumed.119.229187 PMC6954457

[CR42] Rizzo A, Morbelli S, Albano D, Fornarini G, Cioffi M, Laudicella R et al (2024) The Homunculus of unspecific bone uptakes associated with PSMA-targeted tracers: a systematic review-based definition. Eur J Nucl Med Mol Imaging 51:37534 10.1007/s00259-024-06797-5 PMC11445318 38884773

[CR43] Rowe SP, Pienta KJ, Pomper MG, Gorin MA (2018) PSMA-RADS version 1.0: a step towards standardizing the interpretation and reporting of PSMA-targeted PET imaging studies. Eur Urol 73(4):485–487 29132714 10.1016/j.eururo.2017.10.027 PMC6859641

[CR44] Sahlmann CO, Meller B, Bouter C, Ritter CO, Ströbel P, Lotz J et al (2016) Biphasic ⁶⁸Ga-PSMA-HBED-CC-PET/CT in patients with recurrent and high-risk prostate carcinoma. Eur J Nucl Med Mol Imaging 43(5):898–905 26563122 10.1007/s00259-015-3251-y

[CR45] Scarsbrook AF, Bottomley D, Teoh EJ, Bradley KM, Payne H, Afaq A et al (2020) Effect of (18)F-fluciclovine positron emission tomography on the management of patients with recurrence of prostate cancer: results from the FALCON trial. Int J Radiat Oncol Biol Phys 107(2):316–324 32068113 10.1016/j.ijrobp.2020.01.050

[CR46] Silver DA, Pellicer I, Fair WR, Heston WD, Cordon-Cardo C (1997) Prostate-specific membrane antigen expression in normal and malignant human tissues. Clin Cancer Res 3(1):81–85 9815541

[CR47] Trock BJ, Han M, Freedland SJ, Humphreys EB, DeWeese TL, Partin AW, Walsh PC (2008) Prostate cancer-specific survival following salvage radiotherapy vs observation in men with biochemical recurrence after radical prostatectomy. JAMA 299(23):2760–2769 18560003 10.1001/jama.299.23.2760 PMC3076799

[CR48] Witkowska-Patena E, Giżewska A, Dziuk M, Miśko J, Budzyńska A, Walęcka-Mazur A (2020) Diagnostic performance of 18F-PSMA-1007 PET/CT in biochemically relapsed patients with prostate cancer with PSA levels ≤ 2.0 ng/ml. Prostate Cancer Prostatic Dis 23(2):343–348 31780781 10.1038/s41391-019-0194-6

[CR49] Wright GL Jr, Haley C, Beckett ML, Schellhammer PF (1995) Expression of prostate-specific membrane antigen in normal, benign, and malignant prostate tissues. Urol Oncol 1(1):18–28 21224086 10.1016/1078-1439(95)00002-y

