# Supplementary material for: [18F]PSMA-1007 PET for biochemical recurrence of prostate cancer, a comparison with [18F]Fluciclovine
Source: EJNMMI Rep. 2024 Nov 27;8(1):38. doi: 10.1186/s41824-024-00228-2 (PMC11599519; doi:10.1186/s41824-024-00228-2)
Supplement: Supplementary file 1 — Additional file 1 [file 41824_2024_228_MOESM1_ESM.pdf]

Title: [18F]PSMA-1007 PET for biochemical recurrence of prostate cancer, a comparison with [18F]Fluciclovine.

Name authors: Cato C. Loeff, Willemijn van Gemert, Bastiaan M. Privé, Inge M. van Oort, Rick Hermsen, Diederik M. Somford, James Nagarajah, Linda Heijmen, Marcel J.R. Janssen

Corresponding email: cato.loeff@radboudumc.nl

Methods

Imaging procedures

For PET imaging patients received 4 Megabecquerel (MBq)/kg ±10% of [^{18}F]PSMA-1007 and a fixed dose of 370 MBq ±10% of [^{18}F]Fluciclovine (the latter included a preparation of six hours fasting and 24 hours without intensive exercise). Incubation time between PET imaging and intravenous injection of [^{18}F]PSMA-1007 was 90 minutes. Intravenous injection of [^{18}F]Fluciclovine was given on camera and PET imaging started between 3-5 minutes after injection. Both scans were performed on a Siemens Biograph mCT 4-ring scanner. A low-dose CT scan with 3 mm slice thickness was performed for each PET scan.

SUV_{max} assessment

Maximum standard uptake values (SUV_{max}) in lesions were measured on high resolution reconstructed images and complaint to the EARL/EANM guidelines (Aide, Lasnon et al. 2017).

References

Aide, N., et al. (2017). "EANM/EARL harmonization strategies in PET quantification: from daily practice to multicentre oncological studies." Eur J Nucl Med Mol Imaging **44**(Suppl 1): 17-31.
