# Supplementary material for: [18F]PSMA-1007 PET for biochemical recurrence of prostate cancer, a comparison with [18F]Fluciclovine
Source: EJNMMI Rep. 2024 Nov 27;8(1):38. doi: 10.1186/s41824-024-00228-2 (PMC11599519; doi:10.1186/s41824-024-00228-2)
Supplement: Supplementary file 2 — Additional file 2 [file 41824_2024_228_MOESM2_ESM.pdf]

Title: [18F]PSMA-1007 PET for biochemical recurrence of prostate cancer, a comparison with [18F]Fluciclovine.

Name authors: Cato C. Loeff, Willemijn van Gemert, Bastiaan M. Privé, Inge M. van Oort, Rick Hermsen, Diederik M. Somford, James Nagarajah, Linda Heijmen, Marcel J.R. Janssen

Corresponding email: cato.loeff@radboudumc.nl

Assessment form

Scan code:
[^{18}F]PSMA-1007 / [^{18}F]Fluciclovine
Reviewer:
Date of assessment:

Medical Assessment:

- Level of Suspicion (LOS) ; 1. probably benign, 2. uncertain benign, 3. uncertain , 4. uncertain malignant, 5. probably malignant.
- *Substrate CT: lytic/sclerotic/no. Diameter CT lesion: lymph nodes short axis, other long axis.
- Take screenshots (save under own name + scan code) of the suspicious lesions.

Prostate (bed) (T):

Number	Location	Remark	LOS 1-5	CT substrate and diameter (mm)	SUVmax
1					
2					

Pelvic lymph nodes (N):

Number	Location	Remark (including right/left)	LOS 1-5	CT substrate and diameter (mm)	SUVmax
**Local (N)**					
	a. External iliac				
	Presacral				
	Obturatoriusloge				
	a. Internal iliac				
	Mesorectaal				

Distant lymph nodes (M1a):

	a. Iliaca communis				
	Para-aortic				
	Other abdominal focus				
	Aperture to neck				

Skeletal lesions (M1b):

Number	Location	Remark	LOS 1-5	CT substrate and diameter (mm)	SUVmax
1					
2					
3					

Visceral lesions (M1c):

Number	Location	Remark	LOS 1-5	CT substrate and diameter (mm)	SUVmax
1					

Distant lesions (M1):

Number	Location	Remark	LOS 1-5	CT substrate and diameter (mm)	SUVmax
1					

Overall scan quality, visual:

Scan assessable	YES / NO	(circle correct answer)
	**Rating 1-3**	**Remarks**
Overall impression<br>1-3 (poor, fair, good)		
If poor, cause:		
Signal-to-noise ratio<br>1-3 (poor, fair, good)		
Fluciclovine: increased uptake in muscles<br>1-3 (a lot, some, none)		
Both tracers: increased uptake in background<br>1-3 (much, some, none)		
