# Supplementary material for: [18F]PSMA-1007 PET for biochemical recurrence of prostate cancer, a comparison with [18F]Fluciclovine
Source: EJNMMI Rep. 2024 Nov 27;8(1):38. doi: 10.1186/s41824-024-00228-2 (PMC11599519; doi:10.1186/s41824-024-00228-2)
Supplement: Supplementary file 3 — Additional file 3 [file 41824_2024_228_MOESM3_ESM.pdf]

Title: [18F]PSMA-1007 PET for biochemical recurrence of prostate cancer, a comparison with [18F]Fluciclovine.

Name authors: Cato C. Loeff, Willemijn van Gemert, Bastiaan M. Privé, Inge M. van Oort, Rick Hermsen, Diederik M. Somford, James Nagarajah, Linda Heijmen, Marcel J.R. Janssen

Corresponding email: cato.loeff@radboudumc.nl

**Consensus scoring form**

Scan code:
[$^{18}$F]PSMA-1007 / [$^{18}$F]Fluciclovine
Date of consensus reading:
Reader 1 (R1)
Reader 2 (R2)
Reader 3 (R3)

**Explanation:**
In the column by reader: the number of the lesion and the LOS as described on the reader's own assessment form. If the lesion is not described by the reader:

**Conclusion, overall consensus:**

**Identifiable substrate found:** YES / NO (circle correct answer)

| **Location** | **Pos/Neg** | **LOS** | **Specific location** | **SUVmax** | **Suspect substrate yes/no** |
|---|---|---|---|---|---|
| Prostate (bed) (T) | | | | | |
| Pelvic lymph nodes local right (N) | | | | | |
| Pelvic lymph nodes local left (N) | | | | | |
| Distant lymph nodes (M1a) | | | | | |
| Skeletal lesions (M1b) | | | | | |
| Visceral lesions (M1c) | | | | | |
| Other lesions (M1x) | | | | | |

**Prostate (bed) (T):**

| nr | Location | R1 | | R2 | | R3 | | Consensus Pos/Neg | SUVmax |
|---|---|---|---|---|---|---|---|---|---|
| | | nr | LOS | nr | LOS | nr | LOS | | |
| 1 | | | | | | | | | |
| 2 | | | | | | | | | |
| | | | | | | | | | |

**Other distant lesions (M1):**

| Number | Location | Remark | R1 | | R2 | | R3 | | Consensus | SUVmax |
|---|---|---|---|---|---|---|---|---|---|---|
| | | | nr | LOS | nr | LOS | nr | LOS | Pos/neg | |
| 1 | | | | | | | | | | |
| | | | | | | | | | | |

**Lymph nodes:**

| Number | Location | Remark (incl. left/right) | R1 | | R2 | | R3 | | Consensus Pos/Neg | SUV max |
|---|---|---|---|---|---|---|---|---|---|---|
| | | | nr | LOS | nr | LOS | nr | LOS | | |
| **Local (N)** | | | | | | | | | | |
| | a. External iliac | | | | | | | | | |
| | | | | | | | | | | |
| | Presacral | | | | | | | | | |
| | | | | | | | | | | |
| | Obturatoriusloge | | | | | | | | | |
| | | | | | | | | | | |
| | a. Internal iliac | | | | | | | | | |
| | | | | | | | | | | |
| | Mesolectal | | | | | | | | | |
| | | | | | | | | | | |
| | | | | | | | | | | |
| | | | | | | | | | | |
| **Distant lymph nodes (M1a)** | | | | | | | | | | |
| | a. Iliaca communis | | | | | | | | | |
| | | | | | | | | | | |
| | Para-aortic | | | | | | | | | |
| | | | | | | | | | | |
| | Inguinal | | | | | | | | | |
| | | | | | | | | | | |
| | Other abdominal focus | | | | | | | | | |
| | | | | | | | | | | |
| | Aperture to neck | | | | | | | | | |

**Skeletal lesions (M1b):**

| Number | Location | Remark | R1 | | R2 | | R3 | | Consensus Pos/Neg | SUVmax |
|---|---|---|---|---|---|---|---|---|---|---|
| | | | nr | LOS | nr | LOS | nr | LOS | | |
| 1 | | | | | | | | | | |
| 2 | | | | | | | | | | |
| 3 | | | | | | | | | | |
| | | | | | | | | | | |

**Visceral lesions (M1c):**

| Number | Location | Remark | R1 | | R2 | | R3 | | Consensus | SUVmax |
|---|---|---|---|---|---|---|---|---|---|---|
| | | | nr | LOS | nr | LOS | nr | LOS | Pos/neg | |
| 1 | | | | | | | | | | |
| | | | | | | | | | | |
