# Supplementary material for: [18F]PSMA-1007 PET for biochemical recurrence of prostate cancer, a comparison with [18F]Fluciclovine
Source: EJNMMI Rep. 2024 Nov 27;8(1):38. doi: 10.1186/s41824-024-00228-2 (PMC11599519; doi:10.1186/s41824-024-00228-2)
Supplement: Supplementary file 4 — Additional file 4 [file 41824_2024_228_MOESM4_ESM.pdf]

Title: [18F]PSMA-1007 PET for biochemical recurrence of prostate cancer, a comparison with [18F]Fluciclovine.

Name authors: Cato C. Loeff, Willemijn van Gemert, Bastiaan M. Privé, Inge M. van Oort, Rick Hermsen, Diederik M. Somford, James Nagarajah, Linda Heijmen, Marcel J.R. Janssen

Corresponding email: cato.loeff@radboudumc.nl

Table 4. PET/CT characteristics.

Injected activity, MBq	
[^{18}F]PSMA-1007	345.3 ±8.3
[^{18}F]Fluciclovine	379.8 ±3.2
First [^{18}F]PSMA-1007, n (%)	39 (78%)
First [^{18}F]Fluciclovine, n (%)	11 (22%)

Data are mean (±SD) or n (%).
