# Supplementary material for: [18F]PSMA-1007 PET for biochemical recurrence of prostate cancer, a comparison with [18F]Fluciclovine
Source: EJNMMI Rep. 2024 Nov 27;8(1):38. doi: 10.1186/s41824-024-00228-2 (PMC11599519; doi:10.1186/s41824-024-00228-2)
Supplement: Supplementary file 5 — Additional file 5 [file 41824_2024_228_MOESM5_ESM.pdf]

Title: [18F]PSMA-1007 PET for biochemical recurrence of prostate cancer, a comparison with [18F]Fluciclovine.

Name authors: Cato C. Loeff, Willemijn van Gemert, Bastiaan M. Privé, Inge M. van Oort, Rick Hermsen, Diederik M. Somford, James Nagarajah, Linda Heijmen, Marcel J.R. Janssen

Corresponding email: cato.loeff@radboudumc.nl

Table 5. The number of patients with PET-positive disease (PET-positive lesion(s)) on patient level and on region level for both tracers.

	**[^{18}F]PSMA-1007 PET/CT**	**[^{18}F]Fluciclovine PET/CT**	**p value**
**Detection per patient**			
Overall	34 (68%)	21 (42%)	<0.001
**Detection per region**			
Prostate (bed) (T)	*20 (40%)*	11 (22%)	0.022
Pelvic lymph nodes (N)	*11 (22%)*	10 (20%)	1.000
Distant lymph nodes (M1a)	4 (8%)	1 (2%)	0.250
Skeletal lesions (M1b)	4 (8%)	3 (6%)	1.000
Visceral lesions (M1c)	0 (0%)	0 (0%)	-
Other distant lesions (M1x)	0 (0%)	2 (8%)	0.500

Detection rate (n (%)) calculated for [^{18}F]PSMA-1007 PET/CT and [^{18}F]Fluciclovine PET/CT. Two-sided McNemars test. Significant if $p \leq 0.05$.
