# Supplementary material for: [18F]PSMA-1007 PET for biochemical recurrence of prostate cancer, a comparison with [18F]Fluciclovine
Source: EJNMMI Rep. 2024 Nov 27;8(1):38. doi: 10.1186/s41824-024-00228-2 (PMC11599519; doi:10.1186/s41824-024-00228-2)
Supplement: Supplementary file 6 — Additional file 6 [file 41824_2024_228_MOESM6_ESM.pdf]

Title: [18F]PSMA-1007 PET for biochemical recurrence of prostate cancer, a comparison with [18F]Fluciclovine.

Name authors: Cato C. Loeff, Willemijn van Gemert, Bastiaan M. Privé, Inge M. van Oort, Rick Hermsen, Diederik M. Somford, James Nagarajah, Linda Heijmen, Marcel J.R. Janssen

Corresponding email: cato.loeff@radboudumc.nl

Table 6. Contingency table of the consensus majority reads per-patient.

	**[^{18}F]PSMA-1007 +**	**[^{18}F]PSMA-1007 -**
**[^{18}F]Fluciclovine +**	21	0
**[^{18}F]Fluciclovine -**	13	16

p value <0.001
