# Supplementary material for: [18F]PSMA-1007 PET for biochemical recurrence of prostate cancer, a comparison with [18F]Fluciclovine
Source: EJNMMI Rep. 2024 Nov 27;8(1):38. doi: 10.1186/s41824-024-00228-2 (PMC11599519; doi:10.1186/s41824-024-00228-2)
Supplement: Supplementary file 7 — Additional file 7 [file 41824_2024_228_MOESM7_ESM.pdf]

Title: [18F]PSMA-1007 PET for biochemical recurrence of prostate cancer, a comparison with [18F]Fluciclovine.

Name authors: Cato C. Loeff, Willemijn van Gemert, Bastiaan M. Privé, Inge M. van Oort, Rick Hermsen, Diederik M. Somford, James Nagarajah, Linda Heijmen, Marcel J.R. Janssen

Corresponding email: cato.loeff@radboudumc.nl

Table 7. Contingency tables of the consensus majority reads per-region.

Table 7a. Prostate (bed) (T).

	**[^{18}F]PSMA-1007 +**	**[^{18}F]PSMA-1007 -**
**[^{18}F]Fluciclovine +**	9	2
**[^{18}F]Fluciclovine -**	11	28

p value 0.022

Table 7b. Pelvic lymph nodes (N).

	**[^{18}F]PSMA-1007 +**	**[^{18}F]PSMA-1007 -**
**[^{18}F]Fluciclovine +**	9	1
**[^{18}F]Fluciclovine -**	2	38

p value 1.000

Table 7c. Distant lymph nodes (M1a).

	**[^{18}F]PSMA-1007 +**	**[^{18}F]PSMA-1007 -**
**[^{18}F]Fluciclovine +**	1	0
**[^{18}F]Fluciclovine -**	3	46

p value 0.250

Table 7d. Skeletal lesions (M1b).

	**[^{18}F]PSMA-1007 +**	**[^{18}F]PSMA-1007 -**
**[^{18}F]Fluciclovine +**	3	0
**[^{18}F]Fluciclovine -**	1	46

p value 1.000

Table 7e. Visceral lesions (M1c).

	**[^{18}F]PSMA-1007 +**	**[^{18}F]PSMA-1007 -**
**[^{18}F]Fluciclovine +**	0	0
**[^{18}F]Fluciclovine -**	0	50

p value -

Table 7f. *Other distant* lesions (M1x).

	**[^{18}F]PSMA-1007 +**	**[^{18}F]PSMA-1007 -**
**[^{18}F]Fluciclovine +**	0	2
**[^{18}F]Fluciclovine -**	0	48

p value 0.500
