# Supplementary material for: [18F]PSMA-1007 PET for biochemical recurrence of prostate cancer, a comparison with [18F]Fluciclovine
Source: EJNMMI Rep. 2024 Nov 27;8(1):38. doi: 10.1186/s41824-024-00228-2 (PMC11599519; doi:10.1186/s41824-024-00228-2)
Supplement: Supplementary file 8 — Additional file 8 [file 41824_2024_228_MOESM8_ESM.pdf]

Title: [18F]PSMA-1007 PET for biochemical recurrence of prostate cancer, a comparison with [18F]Fluciclovine.

Name authors: Cato C. Loeff, Willemijn van Gemert, Bastiaan M. Privé, Inge M. van Oort, Rick Hermsen, Diederik M. Somford, James Nagarajah, Linda Heijmen, Marcel J.R. Janssen

Corresponding email: cato.loeff@radboudumc.nl

Table 8. Detection rates per-patient and per-region stratified by PSA value for [^{18}F]PSMA-1007 PET/CT and [^{18}F]Fluciclovine PET/CT. Same results are presented in figure 2.

	**PSA value (µg/L)**	**[^{18}F]PSMA-1007 PET/CT**	**[^{18}F]Fluciclovine PET/CT**	**p value**
**Detection per patient**				
Overall	0.2-0.5 (n=28)	17 (60.7%)	7 (25%)	0.002
	≥0.5 (n=22)	17 (77.3%)	14 (63.6%)	0.250
**Detection per region**				
Prostate (bed)	0.2-0.5	10 (35.7%)	2 (7.1%)	0.021
	≥0.5	10 (45.5%)	9 (40.9%)	1.000
Pelvic lymph nodes	0.2-0.5	4 (14.3%)	4 (14.3%)	1.000
	≥0.5	7 (31.8%)	8 (36.4%)	1.000
Distant lymph nodes	0.2-0.5	1 (3.6%)	0 (0%)	1.000
	≥0.5	3 (13.6%)	1 (4.5%)	0.500
Skeletal lesions	0.2-0.5	2 (7.1%)	1 (3.6%)	1.000
	≥0.5	2 (9.1%)	2 (9.1%)	1.000
Visceral lesions	0.2-0.5	0 (0%)	0 (0%)	-
	≥0.5	0 (0%)	0 (0%)	-
Other distant lesions	0.2-0.5	0 (0%)	0 (0%)	-
	≥0.	0 (0%)	2 (9.1%)	0.500

Detection rate (n (%)) calculated for [^{18}F]PSMA-1007 PET/CT and [^{18}F]Fluciclovine PET/CT, stratified for PSA value (0.2-0.5 µg/L (n=28) vs ≥0.5 µg/L (n=22)). Two-sided McNemars test. Significant if p≤0.05.
