# Supplementary material for: [18F]PSMA-1007 PET for biochemical recurrence of prostate cancer, a comparison with [18F]Fluciclovine
Source: EJNMMI Rep. 2024 Nov 27;8(1):38. doi: 10.1186/s41824-024-00228-2 (PMC11599519; doi:10.1186/s41824-024-00228-2)
Supplement: Supplementary file 9 — Additional file 9 [file 41824_2024_228_MOESM9_ESM.pdf]

Title: [18F]PSMA-1007 PET for biochemical recurrence of prostate cancer, a comparison with [18F]Fluciclovine.

Name authors: Cato C. Loeff, Willemijn van Gemert, Bastiaan M. Privé, Inge M. van Oort, Rick Hermsen, Diederik M. Somford, James Nagarajah, Linda Heijmen, Marcel J.R. Janssen

Corresponding email: cato.loeff@radboudumc.nl

Table 9. Detailed reads per-region of three readers, stratified for PSA value.

Prostate (bed) (T)						Pelvic lymph nodes (N)						Distant lymph nodes (M1a)						Skeletal lesions (M1b)						Visceral lesions (M1c)						Other distant lesions (M1x)						PSA
PSMA-1007			Fluciclovine			PSMA-1007			Fluciclovine			PSMA-1007			Fluciclovine			PSMA-1007			Fluciclovine			PSMA-1007			Fluciclovine			PSMA-1007			Fluciclovine			
**R1**	R2	R3	R1	R2	R3	R1	R2	R3	R1	R2	R3	R1	R2	R3	R1	R2	R3	R1	R2	R3	R1	R2	R3	R1	R2	R3	R1	R2	R3	R1	R2	R3	R1	R2	R3	
0	0	1	0	0	0	0	0	0	0	0	0	0	0	0	0	0	0	0	0	0	0	0	0	0	0	0	0	0	0	0	0	0	0	0	0	,20
0	0	2	0	2	0	0	0	0	0	0	0	0	0	0	0	0	0	0	0	0	0	0	0	0	0	0	0	0	0	0	0	0	0	0	0	,20
0	0	1	0	0	0	0	0	0	0	0	0	0	0	0	0	0	0	0	0	0	0	0	0	0	0	0	0	0	0	0	0	0	0	0	0	,20
0	0	1	0	0	0	0	0	0	0	0	0	0	0	0	0	0	0	2	2	0	2	0	0	0	0	0	0	0	0	0	0	0	0	0	0	,21
0	0	0	0	0	0	0	0	0	0	0	0	1	1	1	0	0	0	0	0	0	0	0	0	0	0	0	0	0	0	0	0	0	0	0	0	,21
0	0	0	0	0	0	0	0	2	0	0	0	0	0	0	0	0	0	2	2	0	0	0	0	0	0	0	0	0	0	0	0	0	0	0	0	,22
0	0	1	0	0	0	0	0	0	0	0	0	0	0	1	0	0	0	0	0	0	0	0	0	0	0	0	0	0	0	0	0	0	0	0	0	,23
0	0	0	0	0	0	0	0	0	0	0	0	0	0	0	0	0	0	0	1	2	0	0	0	0	0	0	0	0	0	0	0	0	0	0	0	,26
0	0	1	0	0	0	0	0	0	0	0	0	0	0	0	0	0	0	0	2	0	0	0	0	0	0	0	0	0	0	0	0	0	0	0	0	,26
0	0	1	0	0	0	0	0	0	0	0	0	0	0	0	0	0	0	0	0	0	0	0	0	0	0	0	0	0	0	0	0	0	0	0	0	,27
0	0	0	0	0	1	1	1	1	2	0	1	0	0	0	0	0	0	0	0	0	0	0	0	0	0	0	0	0	0	0	0	0	0	0	0	,27
0	1	0	0	0	0	0	0	2	0	0	2	0	0	0	0	0	0	0	0	0	0	0	0	0	0	0	0	0	0	0	0	0	0	0	0	,28
0	0	0	0	0	0	0	0	2	0	0	0	0	0	0	0	0	0	0	0	0	0	0	0	0	0	0	0	0	0	0	0	0	0	0	0	,30
0	0	0	0	0	0	0	0	0	0	0	0	0	0	0	0	0	0	1	1	1	1	1	1	0	0	0	0	0	0	0	0	0	0	0	0	,30
1	0	1	1	0	0	0	0	0	0	0	0	0	0	0	0	0	0	0	0	0	0	0	0	0	0	0	0	0	0	0	0	0	0	0	0	,30
0	0	1	0	0	0	0	0	0	0	0	0	0	0	0	0	0	0	0	0	0	0	0	0	0	0	0	0	0	0	0	0	0	0	0	0	,30
0	0	0	0	0	0	0	0	0	0	0	0	0	0	0	0	0	0	0	0	0	0	0	0	0	0	0	0	0	0	0	0	0	0	0	0	,30
0	0	0	0	0	0	0	0	0	0	0	0	0	0	0	0	0	0	0	0	0	0	0	0	0	0	0	0	0	0	0	0	0	0	0	0	,30
1	1	2	0	0	0	0	2	0	0	0	0	0	0	0	0	0	0	0	0	0	0	2	0	0	0	0	0	0	0	0	0	0	0	0	0	,30
0	0	0	0	0	0	0	0	1	1	0	1	0	0	0	0	0	0	0	0	0	0	0	0	0	0	0	0	0	0	0	0	0	0	0	0	,32
1	1	1	0	0	2	0	0	0	2	0	1	0	0	0	0	0	0	2	0	0	0	0	0	0	0	0	0	0	0	0	0	0	0	0	0	,33
0	0	0	0	0	0	1	1	1	1	2	1	0	0	0	0	0	0	0	2	0	1	0	0	0	1	0	0	0	0	0	0	0	0	0	0	,35
0	0	0	2	2	1	0	0	0	0	0	0	0	0	0	0	0	0	1	1	0	0	0	0	0	0	0	0	0	0	0	0	0	0	0	0	,36
0	0	0	0	0	0	1	1	1	0	0	0	0	0	0	0	0	0	0	0	0	0	0	0	0	0	0	0	0	0	0	0	0	0	0	0	,37
0	0	0	0	0	0	0	0	0	0	0	0	0	0	0	0	0	0	1	2	2	0	0	0	0	0	0	0	0	0	0	0	0	0	0	0	,38
0	0	0	0	0	0	0	0	0	0	0	0	0	0	0	0	0	0	0	0	0	0	0	0	0	0	0	0	0	0	0	0	0	0	0	0	,38
2	1	1	0	0	0	0	0	0	0	0	0	0	0	0	0	0	0	0	0	0	0	0	0	0	0	0	0	0	0	0	0	0	0	0	0	,40
2	1	1	0	0	0	0	0	0	0	0	0	0	0	0	0	0	0	1	0	0	0	0	0	0	0	0	0	0	0	0	0	0	0	0	0	,43
0	0	0	0	0	0	0	0	0	0	0	0	0	0	0	0	0	0	0	1	0	0	0	0	0	0	0	0	0	0	0	0	0	0	0	0	,50
0	0	0	0	0	0	0	0	0	0	0	0	0	0	0	0	0	0	0	0	0	0	0	0	0	0	0	0	0	0	0	0	0	0	0	0	,50
1	1	1	0	0	0	0	0	0	0	0	0	0	0	0	1	1	0	0	0	0	0	0	0	0	0	0	0	0	0	0	0	0	0	0	0	,74

0	0	0	0	0	0	1	1	1	0	0	1	1	1	1	0	2	0	0	0	0	0	0	0	0	0	0	0	2	0	0	0	0	0	0	0	,80
0	0	0	0	0	0	1	1	1	1	1	1	0	0	0	0	0	0	0	0	0	0	0	0	0	0	0	0	0	0	0	0	0	0	0	0	,82
0	0	0	0	0	0	0	0	0	0	0	0	0	0	0	0	0	0	0	0	0	0	0	0	0	0	0	0	0	0	0	0	0	0	0	0	,83
1	1	1	1	0	1	0	0	0	0	0	0	0	0	0	0	0	0	0	0	0	0	0	0	0	0	0	0	0	0	0	0	0	0	0	0	1,10
0	0	0	2	0	0	1	0	0	1	1	1	0	0	0	0	0	0	1	1	0	0	0	0	0	0	0	0	2	0	0	0	0	0	0	0	1,20
0	0	1	0	0	0	0	0	0	0	0	0	0	0	0	0	0	0	0	2	0	0	0	0	0	0	0	0	0	0	0	0	0	0	0	0	1,30
2	2	1	0	0	1	0	0	0	0	0	0	0	0	0	0	0	0	0	0	0	0	0	0	0	0	0	0	0	0	0	0	0	0	0	0	1,50
0	0	0	2	2	0	1	2	1	0	0	0	0	0	0	0	0	0	0	0	0	0	0	0	2	0	0	0	0	0	0	0	0	0	0	0	1,70
0	0	0	0	0	0	1	1	1	1	1	1	0	0	0	0	0	0	0	0	0	0	0	0	0	0	0	0	0	0	0	0	0	0	0	0	1,90
0	0	0	0	0	0	1	1	1	0	0	1	1	1	1	2	0	1	2	0	0	0	0	0	0	0	0	0	0	0	0	0	0	0	0	0	2,04
1	1	1	1	1	1	0	0	0	0	0	0	0	0	0	0	0	0	0	0	0	0	0	0	0	0	0	0	0	0	0	0	0	0	0	0	2,40
1	1	1	1	1	1	0	0	0	0	0	0	0	0	0	0	0	0	0	0	0	0	0	0	0	0	0	0	0	0	0	0	0	0	0	0	2,70
1	1	1	1	2	1	0	0	0	0	0	0	0	0	0	0	0	0	1	1	1	1	1	1	0	0	0	0	0	0	0	0	0	0	0	0	3,00
1	1	1	0	0	1	0	0	0	0	0	0	0	0	0	2	2	0	0	0	0	0	0	0	0	0	0	0	0	0	0	0	0	0	0	0	3,60
0	0	0	0	0	0	0	0	0	0	0	0	0	0	0	0	0	0	0	0	0	0	0	0	0	0	0	0	0	0	0	0	0	0	0	0	3,60
2	0	0	0	2	2	1	1	1	2	1	1	2	0	1	0	0	0	0	0	0	0	0	0	0	0	0	0	0	0	0	0	0	0	0	0	3,80
1	1	1	1	1	1	0	0	0	0	0	0	0	0	0	0	0	0	0	0	0	0	0	2	0	0	0	0	0	0	0	0	0	0	0	0	3,80
1	1	1	1	0	1	0	0	0	0	0	0	0	0	0	0	0	0	0	0	0	0	0	0	0	0	0	0	0	0	0	0	0	0	0	0	4,10
1	1	1	1	1	1	0	0	0	0	0	0	0	0	0	0	0	0	1	1	1	0	1	1	0	0	0	0	0	0	0	0	0	0	0	0	4,60

R=reader, PSMA-1007= [^{18}F]PSMA-1007, Fluciclovine= [^{18}F]Fluciclovine. 0 = negative PET/CT (LOS 1-2), 1 = positive PET/CT (LOS 4-5), and 2 = unsure (LOS 3) PET/CT.
