# Supplementary material for: [18F]PSMA-1007 PET for biochemical recurrence of prostate cancer, a comparison with [18F]Fluciclovine
Source: EJNMMI Rep. 2024 Nov 27;8(1):38. doi: 10.1186/s41824-024-00228-2 (PMC11599519; doi:10.1186/s41824-024-00228-2)
Supplement: Supplementary file 10 — Additional file 10 [file 41824_2024_228_MOESM10_ESM.pdf]

Title: [18F]PSMA-1007 PET for biochemical recurrence of prostate cancer, a comparison with [18F]Fluciclovine.

Name authors: Cato C. Loeff, Willemijn van Gemert, Bastiaan M. Privé, Inge M. van Oort, Rick Hermsen, Diederik M. Somford, James Nagarajah, Linda Heijmen, Marcel J.R. Janssen

Corresponding email: cato.loeff@radboudumc.nl

Table 10. Follow-up treatment (median follow-up of 28.5 months).

**Follow-up treatment**	
Radiation therapy prostate (bed)	11 (22%)
Radiation therapy prostate (bed) and lymph node	3 (6%)
Radiation therapy lymph node	3 (6%)
Radiation therapy lymph node + Pelvic lymph node dissection	1 (2%)
Radiation therapy distant	3 (3%)
Radiation therapy prostate (bed) + Androgen deprivation therapy*	4 (8%)
Radiation therapy prostate (bed) + Androgen deprivation therapy	1 (2%)
Radiotherapy lymph node + Androgen deprivation therapy*	1 (2%)
Radiation therapy prostate (bed) and lymph node + Androgen deprivation therapy*	2 (4%)
Pelvic lymph node dissection	1 (2%)
Androgen deprivation therapy	7 (14%)
Brachytherapy	1 (2%)
Active surveillance	8 (16%)

*Follow-up treatment (n(%)). * Only temporary additional hormonal treatment in adjuvant setting to reduce risk of disease recurrence.*
