# Supplementary material for: [18F]PSMA-1007 PET for biochemical recurrence of prostate cancer, a comparison with [18F]Fluciclovine
Source: EJNMMI Rep. 2024 Nov 27;8(1):38. doi: 10.1186/s41824-024-00228-2 (PMC11599519; doi:10.1186/s41824-024-00228-2)
Supplement: Supplementary file 11 — Additional file 11 [file 41824_2024_228_MOESM11_ESM.pdf]

Title: [18F]PSMA-1007 PET for biochemical recurrence of prostate cancer, a comparison with [18F]Fluciclovine.

Name authors: Cato C. Loeff, Willemijn van Gemert, Bastiaan M. Privé, Inge M. van Oort, Rick Hermsen, Diederik M. Somford, James Nagarajah, Linda Heijmen, Marcel J.R. Janssen

Corresponding email: cato.loeff@radboudumc.nl

Table 11. Lesion validation per region, stratified for PSA value.

	[18F]PSMA-1007 PET/CT				[18F]Fluciclovine PET/CT					
**PSA**	**Scored region**	**+ or -**	**Confirmed region**	**TP/ FP/ TN/ FN**	**Scored region**	**+ or -**	**Confirmed region**	**TP/ FP/ TN/ FN**	**Confirmation procedure**	**Follow-up treatment**
0.20	T	+	T	TP				FN	PSA decrease after local/targeted treatment	Radiation therapy prostate (bed)
0.20	T	+	T	TP				FN	PSA decrease after local/targeted treatment	Radiation therapy prostate (bed)
0.21	M1a	+	M1a	TP				FN	PSA decrease after local/targeted treatment	Radiation therapy distant
0.23	T	+	T	TP				FN	PSA decrease after local/targeted treatment	Radiation therapy prostate (bed) + LN
0.26	T	+	T	TP				FN	PSA decrease after local/targeted treatment	Radiation therapy prostate (bed)
0.27	T	+	T	TP				FN	PSA decrease after local/targeted treatment	Radiation therapy prostate (bed) + LN + ADT*
0.27	N	+	N	TP	N	+	N	TP	PSA decrease after local/targeted treatment	Radiation therapy prostate (bed)
0.28	N	-	N	TN	N	-	N	TN	No PSA decrease after local/targeted treatment	Radiation therapy LN
0.30	M1b	+	M1b	TP	M1b	+	M1b	TP	Progression on follow-up imaging (PSMA PET/CT)	ADT
0.30	T	+	T	FP	T	+	T	FP	Progression on follow-up imaging (PSMA PET/CT) PSA decrease without intervention	Active surveillance
0.30	T	+						FN	PSA decrease after local/targeted treatment	Radiation therapy prostate (bed)
0.32	N	+	N	TP	N	+	N	TP	PSA decrease after local/targeted treatment	Radiation therapy prostate (bed) + LN
0.33	T	+			N	+	N	FP	PSA decrease after local/targeted treatment	Radiation therapy prostate (bed) + ADT*
0.36	M1b	+	M1b	TP				FN	Progression on follow-up imaging (PSMA PET/CT + MRI)	Radiation therapy distant

									PSA decrease after local/targeted treatment	
0.37	N	+	N	TP				FN	PSA decrease after local/targeted treatment	Radiation therapy LN
0.38				TN				TN	Remained negative on follow-up imaging (PSMA PET/CT)	Active surveillance
0.40	T	+	T	*TP*				FN	Progression on follow-up imaging (PSMA PET/CT)	Active surveillance
0.43	T	+	T	TP				FN	Progression on follow-up imaging (PSMA PET/CT)	Active surveillance
0.74	T	+	T	TP				FN	Progression on follow-up imaging (MRI) + PSA decrease after local/targeted treatment	Radiation therapy prostate (bed) + ADT
0.80	N, M1a	+	N, M1a	TP	N, M1x	+	N, M1x	TP	Progression on follow-up imaging (FDG PET/CT)	ADT
0.82	N	+	N	TP	N	+	N	TP	Histology + PSA decrease after local/targeted treatment	Radiation therapy LN + PLND
1.10	T	+	T	TP	T	+	T	TP	Progression on follow-up imaging (MRI) + PSA decrease after local/targeted treatment	Radiation therapy prostate (bed)
1.20	N	+			N	+				ADT
1.50	T	+						FN	Progression on follow-up imaging (MRI) + PSA decrease after local/targeted treatment	Radiation therapy prostate (bed) + LN
1.70	N	+	N	TP				FN	Histopathology + Progression on follow-up imaging (PSMA PET/CT)	PLND
1.90	N	+	N	TP	N	+	N	TP	PSA decrease after local/targeted treatment	Radiation therapy LN
2.00	N, M1a	+			N, M1a	+				ADT
2.70	T	+			T	+				Active surveillance
3.00	T, M1b	+			T, M1b	+				ADT
3.60	T	+			T	+			Progression on follow-up imaging (PSMA PET/CT) PSA decrease after local/targeted treatment	Radiation therapy prostate (bed)
3.80	N, M1a	+			T, N	+				ADT
3.80	T	+	T	TP	T	+	T	TP	PSA decrease after local/targeted treatment	Radiation therapy prostate (bed)
4.10	T	+			T	+			PSA decrease after local/targeted treatment	Brachytherapy
4.60	T, M1b	+	T, M1b	TP	T, M1b	+	T, M1b	TP	Progression on follow-up imaging (PSMA PET/CT)	Not known

Lesion conformation per region. Data is presented for patients with at least one positive or negative scored region with one of the two PET-tracers. Those with no follow-up data available are not presented. Patients with both scans score negative without scored region were FN as a whole, because PSA rise is indicative for recurrent disease. These patients are not presented in the table. One patient, with no negative scored region, remained negative on follow-up imaging and could therefore be scored TN as a whole. Last PSA before scan, scored region (T, N, M1a, M1b, M1x), scored region positive (+) or negative (-), confirmed region (T, N, M1a, M1b, M1x) and confirmation (TP/FP/TN/FN) per tracer, confirmation procedure (based on composite reference score) and follow-up treatment

*are presented. Not all findings per region could be confirmed per region as for example these patients received systemic therapy without previous confirmation of lesions. PSA = prostate-specific antigen, LN = lymph nodes, FDG = Fluorodeoxyglucose, PLND = pelvic lymph node dissection, ADT = androgen deprivation therapy, * Only temporary additional hormonal treatment in adjuvant setting to reduce risk of disease recurrence.*
